# Basic research in orthopedics: South Africa

**DOI:** 10.4103/0019-5413.55970

**Published:** 2009

**Authors:** Shunmugam Govender

**Affiliations:** *Department of Orthopaedic Surgery, Nelson R Mandela Medical School, University of KwaZulu-Natal, Durban-4001, South Africa*

Basic science and clinical research form the very essence and future development of orthopedics. Although tremendous strides have been made in enriching orthopedics, most of the current contributions are from the well-resourced First World countries. Inequalities in health research contribute to inequalities in health. Research should reflect national priorities and focus particularly on common orthopedic pathology in Africa's population, evaluate interventions that aim to strengthen health systems, and should focus on activities to convert knowledge into action. In sub-Saharan Africa, the current sociopolitical and economic conditions militate against sustained meaningful basic science research to address pertinent health care problems prevalent in the continent.^1^ The historical relations between countries due to shared languages and economic or security interests tend to promote contacts between researchers and may provide funding for collaboration. The commission on health research has recommended that at least 2% of national health budgets and 5% of development aid should be invested in health research and on building research capacity. Part of the funds for research should be directed to strengthening the capacity to conduct research, manage research by establishing processes to handle grant funding, review the ethics of proposed research, and develop skills in scientific writing. Africa's researchers, governments, and partners must ensure that knowledge generated from research is acted on to improve health for all. Research must inform health policy as well as medical care.^1^

The capacity to conduct and publish research varies widely across African countries. Practical difficulties include the infrastructure, systems for information and communication, travel, foreign exchange, and safety which may influence the establishment and maintenance of scientific interaction.^1^ Such factors especially in Africa leave the most disadvantaged countries as the least likely to attract investment research. A World Bank report that ranked countries in Africa according to their national health investments and productivity in science and technology noted that South Africa and Egypt did reasonably well, while the rest of Africa appeared at the bottom of the league under scientifically lagging countries. Information exchange is a critical force for international development especially in Africa. The importance of information transfer in medicine is perhaps best exemplified by the previous debate in South Africa regarding the value of antiretroviral drugs in the treatment of HIV infection. Inappropriate scientific advice, lack of political will, and the delay in implementing antiretroviral treatment resulted in increased morbidity and mortality in South Africa. Poor funding from public and private sources remains the most serious limitation to fostering a research culture in basic science in the developing world. Government priorities frequently lie elsewhere (defense and debt repayment). This chronic lack of research support has created impoverished academic and laboratory facilities, which have been worsened by weak institutional and technical support and interest in encouraging research that meets international standards. A fundamental difficulty is that even when research is contemplated, the questions posed may not be focused on the most serious health issues facing the country. Given the poor salary structure in public service, doctors turn to private practice rather than research to supplement meager incomes.^2^ An enormous clinical and teaching workload precludes research. Often, this choice is one of survival for doctors and their families. Rapid private practice development has provided highly accessible clinical services for approximately 7 million of the 47 million people in South Africa. Private practice has become increasingly attractive to health care professionals owing to the wide differential in income and general working conditions between state and private practice. Currently, 31% of specialist posts in South African tertiary-level training hospitals are vacant. The limited number of specialists in academic departments at all levels diminishes the ability to sustain many important departmental functions, the most vulnerable of which is research.^3^

The present worldwide economic crisis severely restricts the availability of clinical and research periodicals. Print online subscriptions and reprint costs are prohibitive. The former UN secretary general Kofi Anan announced the creation of a UN Information Technology Service (UNITES) to provide access to medical information in developing countries. Much more research needs to be aimed at identifying information needs in less developed countries. Collaboration on randomized trials of health information systems might help to promote a culture of writing and create important clinical and research databases. Publishers might also consider producing cost-effective local editions in less developed countries in conjunction with local publishers and creating regional editorial offices to provide a human bridge across continents. Selected articles relevant to the African continent form the African edition of the combined volumes of the Journal of Bone and Joint Surgery. The publishers in South Africa distribute the journal in Southern and East Africa free of charge. International donor funding might be useful in providing or subsidizing the purchase of equipment for libraries to assist online access. The European Association of Science Editors, the Council of Science Editors, the International Committee of Medical Journal Editors, and the World Association of Medical Editors also have an important part to play. These editorial boards can play a pivotal role in encouraging scientific publications from the developing world. These parties could help establish regional databases, such as the already existing African Index Medicus funded from the World Health Organization (WHO), the Association for Health Information, and the Norwegian Development, co-operative agency.

The new millennium is facing a profound threat from tuberculosis (TB) in the African continent. The WHO has recognized that the alarming growth of drug-resistant strains of TB could well become a global phenomenon, threatening the achievement of both health and economic goals. In response to this, millions of dollars have been earmarked internationally for research on new vaccines, improved and faster diagnostic methods, and new drugs for shorter treatment regimen and to combat drug-resistant strains.

Collaboration in research may be a vehicle for strengthening the research capacity in less privileged countries. Collaboration may improve the New Partnership for Africa's Development (NEPAD) which is actively facilitating partnerships within Africa and with the international community. Guidelines on international partnerships in research should help to minimize the risks in research collaboration.^4^–^6^ The South African Orthopaedic Association and orthopedic surgeons from Southern and East Africa formed the East, Central, and Southern African Orthopaedic Association (ECSAOA) in 2000 and member countries include South Africa, Zimbabwe, Zambia, Malawi, Kenya, Mozambique, and Uganda. Through the generous funding from multinational organizations, fellowships are provided in South Africa for colleagues from neighboring countries in basic science and clinical orthopedics. In addition, we have established centers of excellence in the region for undergraduate and postgraduate teaching. Six teams of surgeons from South Africa travel annually to the region to teach and train undergraduate and postgraduate students of orthopedics. An entire day is now dedicated to orthopedics at the annual meeting of the Association of Surgeons of East Africa (ASEA) for instructional courses and the presentation of common orthopedic pathology in the region. The basic science laboratory services in South Africa provide an ideal resource for research especially in HIV, TB, and other diseases which are common in the region. Many sophisticated and highly advanced technological facilities for basic science medical research are available at the Medical Research Council (MRC), Council for Scientific and Industrial Research (CSIR), and South African Institute for Medical Research (SAIMR) and universities within South Africa. The MRC, CSIR, and the SAIMR provide funding for research.^7^

Africa is home to approximately 700 million of the world's population. South Africa is the industrial and educational hub of the African continent. Clinical medicine has been one of the mainstays of the South African research. Although the absolute number of publications has increased during the last two decades, since its highest level of 1063 publications in 1987, the rate of increase has not kept pace with the international growth. During the last two decades, South African scientific output grew at a compounded rate of 2.4% per year. The future is bleak if African leaders and governments fail to address the serious health issues facing the continent.
